# Improvement in Resident Scholarly Output with Implementation of a Scholarly Activity Guideline and Point System

**DOI:** 10.5811/westjem.60346

**Published:** 2023-08-15

**Authors:** Lauren Evans, Sarah Greenberger, Rachael Freeze-Ramsey, Amanda Young, Crystal Sparks, Rawle Seupaul, Travis Eastin, Carly Eastin

**Affiliations:** University of Arkansas for Medical Sciences, College of Medicine, Department of Emergency Medicine, Little Rock, Arkansas

## Abstract

**Introduction:**

Ensuring high-quality scholarly output by graduate medical trainees can be a challenge. Within many specialties, including emergency medicine (EM), it is unclear what constitutes appropriate resident scholarly activity. We hypothesized that the quantity and quality of scholarly activity would improve with a clearer guideline, including a point system for eligible scholarly activities.

**Methods:**

A resident Scholarly Activity Guideline was implemented for EM residents in a university setting. The guideline consists of a point system in which point values, ranging from 1–10, are assigned to various types of scholarly activities. Residents must earn at least 10 points and present their work to meet their scholarly graduation requirement. We tracked scholarly activities for graduates from the classes of 2014–2020, with the guideline being implemented for the class of 2016. In a blind analysis, we compared median total points per resident, mean counts of the Boyer model of scholarship components per resident, and mean counts of significant scholarly output per resident before vs after the guideline was implemented. Significant scholarly output was defined as an implemented protocol, a research project with data collection and analysis, a research abstract presentation, or an oral abstract presentation.

**Results:**

Among 64 residents analyzed, 48 residents used the guideline. We found that median points per resident increased after the guideline was implemented (median, interquartile range: before 7 [7], after 11 [10, 13], *P* = 0.002). Post-guideline scholarly activities were found to represent more of Boyer’s components of scholarship [mean before 0.81 [SD 0.40], mean after 1.52 [SD 0.71], mean difference 0.71, 95% confidence interval [CI] 0.332 ± 1.09, *P* < 0.001. There was no difference in the mean significant scholarly output per resident (mean before 1.38 [SD 1.02], mean after 1.02 [SD 1.00], mean difference 0.35, 95% CI 0.93 ± 0.23, *P* = 0.23).

**Conclusion:**

Implementation of a Scholarly Activity Guideline point system significantly increased the quantity and, by one of two measures, increased the quality of scholarly output in our program. Our point-based guideline successfully incorporated traditional and modern forms of scholarship that can be tailored to resident interests.

Population Health Research Capsule What do we already know about this issue?

*Scholarly activities are a required part of residency training, but defining appropriate scholarship remains a challenge for residency leadership.*

What was the research question?

*Can a novel scholarly activity guideline and point system improve the quality and quantity of resident scholarship?*

What was the major finding of the study?

*Implementation of the point system correlated with improved median scholarly points per resident (before 7 [IQR 7], after 11 [IQR 10, 13], P = 0.002).*

How does this improve population health?

*A clear, concise scholarly activity guideline may allow for improved resident scholarly output, which contributes to the advancement of emergency care.*

## INTRODUCTION

Scholarship has been a fundamental requirement during any residency training since 1994, but executing this requirement can be challenging due to confusion regarding what constitutes scholarship.^1^ It is consistently thought that all trainees should be exposed to the four components of the Boyer model for scholarship: discovery; integration; application; and teaching.^2^^,^^3^ In 2013, the Common Program Requirements for residency programs as published by the Accreditation Council for Graduate Medical Education (ACGME) stated that, “residents must participate in scholarship.”^4^ While there were more specific faculty scholarship expectations, the emergency medicine (EM) requirements did not dictate the type or extent of this requirement for residents. In July 2022, the ACGME added more detailed language to these broad requirements for EM (Table 1), but they can still be difficult for program leadership to execute.^5^

**Table 1.** Evolution of emergency medicine resident scholarship requirements as designated by the ACGME Residency Review Committee for emergency medicine.^4^^,^^5^

2013 Emergency medicine requirements for resident scholarly activities	The curriculum must advance residents’ knowledge of the basic principles of research, including how research is conducted, evaluated, explained to patients, and applied to patient care.
	Residents should participate in scholarly activity.
	The sponsoring institution and program should allocate adequate educational resources to facilitate resident involvement in scholarly activities.
2022 Emergency medicine requirements for resident scholarly activities	Residents must participate in scholarship.
	The curriculum must advance the residents’ knowledge of the basic principles of research, including how research is conducted, evaluated, explained to patients, and applied to patient care.
	At the time of graduation, each resident should demonstrate: • active participation in a research project, or formulation and implementation of an original research project, including funded and non-funded basic science or clinical outcomes research, as well as active participation in an Emergency Medicine emergency department quality improvement project; or, • presentation of grand rounds, posters, workshops, quality improvement presentations, podium presentations, webinars; or, • grant leadership, non-peer-reviewed print/electronic resources, articles or publications, book chapters, textbooks, service on professional committees, or serving as a journal reviewer, journal editorial board member, or editor; or • peer-reviewed publications.

*ACGME*, Accreditation Council for Graduate Medical Education.

Not surprisingly, there has been broad and subjective application of this requirement. For example, in 2015 Geyer et al found that within EM, 39% of programs surveyed required an original research project upon graduation while 61% of programs allowed curricular development projects or evidence-based reviews as an alternative to a traditional research project with an associated peer-reviewed manuscript.^1^ In an attempt to address this issue, in 2018 representatives from two national EM groups published recommendations based on survey data, suggesting appropriate types of scholarship for EM residents to provide more structure to the scholarship requirement.^6^ However, these recommendations have been cited as being too strict and not taking into account more modern forms of scholarship such as social media, podcasts, or online curricula, which are now recognized as fulfilling the Boyer components of scholarship.^7^^,^^8^ Since academic institutions are increasingly considering more varied types of scholarship in the faculty promotion process, it is reasonable to similarly allow residents to participate in traditional and nontraditional activities based on their interests.

In addition to the historical variation in programs’ interpretations of scholarship requirements, enforcing a single standard for the scholarly activity requirement may not be appropriate, as many trainees do not plan to pursue a career in academic medicine. Trainees often equate research or scholarly activity with academia, which can result in diminished engagement in these projects if they intend to pursue a different career path. Further complicating the scholarly process, many residents have difficulty completing original research projects due to lack of mentorship or institutional support for data collection or statistical analysis.^1^^,^^9^ It is, therefore, not surprising that inadequate scholarship has led to a high rate of Review Committee (RC) citations since the scholarship requirement was introduced in 1994.^1^^,^^9^

In 2018, a meta-analysis evaluating graduate medical education (GME) scholarship initiatives found that, while no specific strategy was more effective at increasing trainee publications, there was a significant increase in publications following the implementation of any initiative. The authors concluded that a “culture of emphasis on resident scholarship” was the critical factor in increasing scholarly production among trainees.^10^ Several other strategies have also been described that may improve resident scholarly output, with the most effective being providing dedicated time for scholarship, having a research curriculum, a defined scholarship requirement, requiring a presentation at a research day, or a combination of these.^11^ However, the optimal approach remains unclear.

## OBJECTIVE

The purpose of this innovation was to improve the quantity and quality of scholarship by providing a well-defined scholarly activity framework for our trainees while simultaneously incorporating options for a variety of traditional and modern scholarly activities.

## CURRICULAR DESIGN

### Development of the Scholarly Activity Guideline

After a literature review in 2014, we used existing recommendations for scholarly output in an EM training program along with a previously published unique scholarship point system to create a Scholarly Activity Guideline (SAG, Supplemental Material).^3^^-^^5^^,^^12^^,^^13^ While not a proven method, we chose this point system based on the novelty of the idea and the inclusion of a wide variety of options for scholarship. The point system was tailored to our program’s goals for fulfilling the scholarship requirement. It provides multiple options for scholarship with associated point values ranging from 1–10 (Table 2).

**Table 2.** The original scholarly activity point system, created in 2014, adapted from Seehusen et al.^a^

Type of scholarly activity	Points
IRB-approved project completed with manuscript submitted to a peer-reviewed journal	≥10
Submission of a manuscript describing a case series, systematic review, or meta-analysis	≥10
Presentation of a poster or oral presentation at a regional, national, or international conference	5
Publication of a book chapter or section	10
Non IRB-approved quality-improvement (QI) project completed and results shared with peers	7
Initiation of IRB-approved research or QI project but project still ongoing at time of graduation	8 – 10
Submission of a grant for intramural or extramural funding (with IRB approval)	10
Creation and maintenance of an online teaching tool	5
Publication of a letter to the editor in a peer-reviewed medical journal	3 – 5
Creation of simulation case for simulation curriculum (not published vs published)	3 – 10
Submission to peer-reviewed journal or national conference of a series of interesting cases (ie, visual diagnosis cases or photo competition)	3.5
Publications for the lay public, such as newspaper articles, on medical topics	3
Participation on a national committee	5
Critically appraised topic write-up and submission to journal	5

a Table adapted from Seehusen DA, Asplund CA, Friedman M. A point system for resident scholarly activity. *Fam Med*. 2009 Jul–Aug;41(7):467–9. PMID: 19582627.

*IRB*, institutional review board.

The first version of SAG required that residents obtain a minimum of 10 points on the scholarly point system, attend one department research meeting yearly, complete evidence-based learning modules, and present their work at our annual departmental Scholar’s Day. The meeting and module requirements have since been removed as they are addressed elsewhere in the residency curriculum. The guideline includes a suggested timeline for completion to assist in keeping residents on schedule. Finally, it includes an idea form which serves as a guide for in-person project discussions with members of the research committee. Residents are required to obtain approval from the Research Committee for any scholarship for which they wish to obtain points, but generally they can choose any combination of activities from the guideline to achieve the goal of 10 points. If there is a project that does not fit well within the options listed, residents can present their idea for consideration to the Research Committee and, if approved, the committee assigns points by consensus.

The SAG was implemented in August 2014 and was first applied to the graduating class of 2016 as well as all subsequent residents. This time gap allowed the intern class of 2014 two academic years to meet their requirements. As a matter of background, our EM residency is a longstanding three-year program located at a Level 1 trauma and tertiary care hospital in the South Central United States. Prior to the introduction of the SAG, there was a scholarship requirement for all residents without a specific curriculum or guide defining what types of projects were appropriate. Over the seven years of this data analysis, our residency complement increased from eight to 10 residents per year. We also added several additional faculty, including one research-focused faculty member.

For quality improvement purposes, the guideline has been reviewed periodically and adjustments to it have been made when necessary. For example, we removed the requirement to attend a research meeting as they were not found to be high yield. To avoid diminishing educational value, restrictions on case reports were also added to prevent residents from only completing case reports during their training. Lastly, the category of abstracts for the *Journal of Emergency Medicine* was added, as one of the program’s faculty is the section editor for the Abstracts section of the journal and allows residents to contribute as authors.

### Methods for Analysis of the Scholarly Activity Guideline

As part of routine residency documentation, scholarly activity output for all residents is recorded in real time. In June 2020 we abstracted all recorded scholarship for graduates between 2014-2020 from each graduate’s exit letter to assess the improvement in scholarly output related to the introduction of the new guideline. While all projects after 2015 had points assigned to them at the time they were completed, three authors (SG, RFR, AY) who were not members of the Research Committee independently assigned points for the pre-SAG cohort and re-measured point totals for each resident in the post-SAG group to ensure consistency and reduce bias. To further reduce bias, two authors (TE, CE) removed the name and graduation year of the resident and replaced the project title with a project type. This blinded list was then randomized and entered into RedCap, an electronic data capture tool hosted at University of Arkansas for Medical Sciences for rating. The same authors (SG, RFR, AY) also analyzed the quality of each resident’s output based on how many, if any, of the Boyer components of scholarship were represented. Therefore, our two primary outcomes were the comparison of the median number of points per resident and the mean number of the Boyer components of scholarship present per resident, before and after implementation of the SAG.

Although the consensus document regarding appropriate scholarly output for EM residents by Kane et al was published several years after implementation of our guideline, we wanted to incorporate these criteria into our analysis as well.^6^ In addition to recommending that programs maintain an archive in residency record files, Kane et al proposed four other primary elements of resident scholarship: a developed and implemented protocol; a research paper with a hypothesis, collected and analyzed data, and a conclusion; a research abstract presentation; or an oral research presentation. The consensus authors stated these were recommendations rather than requirements, and while those options were considered to be best practices, they still believed that program directors could accept alternative types of scholarship.^6^ As a pre-planned secondary analysis, our three raters (SG, RFR, AY) also rated which projects met at least one of the consensus criteria for scholarship, which we defined as “significant scholarly output.”

The university’s institutional review board (IRB) did not consider this review to be human subjects research and, therefore, did not require IRB oversight. We used descriptive statistics where appropriate. The median value among the three raters was used as the final count for each variable. We treated our scholarly activity point variable, as ordinal as different activities could achieve differing point values. Counts of the Boyer components and significant scholarly output were treated as continuous. Categorical variables were compared using chi-squared tests (or the Fisher exact test if counts were rare), ordinal variables were compared using the Mann-Whitney U test, and continuous variables were compared using independent *t*-test. We calculated inter-rater reliability (Fleiss Kappa) to assess the agreement between the three raters. Analyses were performed using SPSS Statistics for Macintosh version 28.0 (IBM Corp, Armonk, NY).

## IMPACT

### Analysis of Scholarly Output

**Figure 1.**
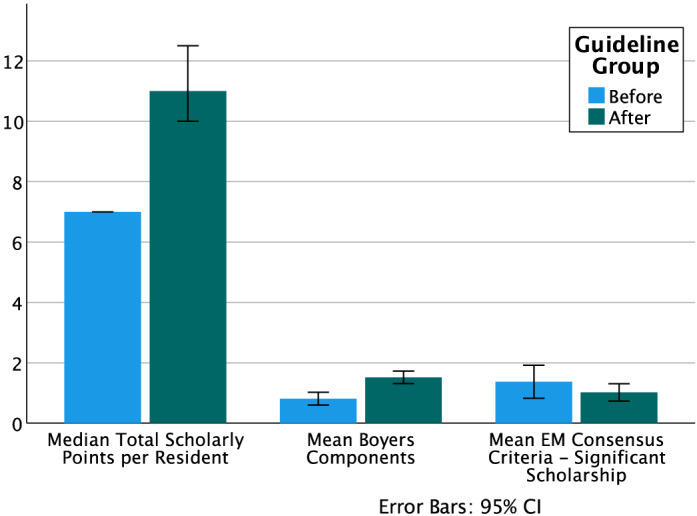
Outcomes before vs after scholarly guideline. *EM*, emergency medicine.

Sixty-four residents graduated in the period studied, producing 676 scholarly points. Before the guideline, only one of 16 (6.25%) residents would have met the minimum point requirement (Table 3). Since the SAG was implemented, 40/48 (83.3%) residents in the classes of 2016–2020 met their scholarly point requirements based on the blinded review. Total points per resident increased significantly after implementation of the guideline (median 7 points per resident [interquartile range (IQR) 7, 7] before vs 11 points per resident after [IQR 10, 13], *P* < 0.002). See Figure 1 for graphical representation of all outcomes. Of note, 54.2% (26/48) of residents in the post-guideline period ended their training with more points than the minimum required, compared with 6.25% (1/16) before (relative risk [RR] 2.05, 95% confidence interval [CI] 1.47–2.85, *P* < 0.001).

**Table 3.** Scholarly output results, before and after.

		Guideline group			
		Before		After	
		Count	%	Count	%
Successfully achieved minimum points	≥10 points	1	6.3%	40	83.3%
	<10 points	15	93.8%	8	16.7%
Boyer components present	0	3	18.8%	2	4.2%
	1	13	81.3%	23	47.9%
	2	0	0.0%	19	39.6%
	3	0	0.0%	4	8.3%
	4	0	0.0%	0	0.0%
At least one Boyer component present	Yes	13	81.3%	46	95.8%
	No	3	18.8%	2	4.2%
Significant scholarly output present	0	2	12.5%	16	33.3%
	1	9	56.3%	22	45.8%
	2	3	18.8%	3	6.3%
	3	1	6.3%	7	14.6%
	4	1	6.3%	0	0.0%
At least one significant scholarly output	Yes	14	87.5%	31	64.6%
	No	2	12.5%	17	35.4%

Regarding the quality of scholarship, we found at least one of the Boyer components of scholarship in 92% (59/64) of resident portfolios as well as at least one significant scholarly achievement in 71.8% (46/64). When comparing before and after the SAG, we found a significant increase in the mean number of Boyer components (mean before 0.81 [SD 0.40], mean after 1.52 [SD 0.71], mean difference 0.71, 95% CI 0.332–1.09, *P* < 0.001), although the proportion of residents with at least one component did not change (81.25% vs 95.3%, RR = 4.5, 95% CI 0.82–25.47, *P* = 0.10). There was no change in the average number of significant scholarly outputs per resident (mean before 1.38 [SD 1.02], mean after 1.02 [SD 1.00], mean difference −0.35, 95% CI −0.93 – 0.23, *P* = 0.23) or in the proportion of residents with at least one significant scholarly output (87.5% vs 64.58%, RR 0.35, 95% CI 0.09 = −1.36, *P* = 0.12). Overall interrater agreement was measured as moderate (Κ = 0.538, 95% CI 0.499–0.577, *P* < 0.001).

As a natural consequence of seeing more of the Boyer components of scholarship with the SAG, we noticed that the types of scholarship appeared to be more varied as well. In the pre-guideline period, most scholarly activities were local quality improvement projects that resulted in data collection and analysis, but most were only presented locally. Two of these resulted in research abstracts presented at national meetings, and there was one resident who participated in a multicenter, chart review study. In the post-guideline period, residents not only had regional and national research abstract presentations, peer-reviewed manuscripts, and book chapters, but they also published evidence-based reviews, edited online resources for medical students, wrote articles for community newsletters, and recorded educational, evidence-based podcasts. In addition, while participation in national committees was initially a controversial addition to our accepted forms of scholarship, residents who have chosen this option have been highly engaged and were often invited to be authors on subsequent publications or participate in national presentations.

## DISCUSSION

We are excited about the improved resident scholarship we achieved after implementation of the SAG. We believe that having a clearer and more diverse list of options has allowed residents to successfully choose projects in which they would feel engaged while the point requirement has ensured a minimum standard and accountability. Although anecdotal, the faculty members overseeing resident scholarship have noted that the clarity provided by the SAG seems to have reduced frustration on the part of both residents and faculty. While the SAG concept is novel to EM, this point system was adapted from a similar system used in a family medicine residency by Seehusen et al.^12^ Our improvements are congruent with the increase they found in their scholarly output with this approach, describing more presentations, book chapters, and peer-reviewed publications. Since implementation of our guideline, execution of a similar point system has been described in the radiology literature, but no official quantitative analyses have been reported.^14^

We found it interesting that while the total points increased and we found more of the Boyer components of scholarship present after the guideline, we did not see a difference in significant scholarly output as defined as one of the four criteria described by Kane et al in their consensus statement.^6^ While Kane et al’s recommendation was made with input and support from members of several EM organizations, it was primarily crafted by members of the Research Directors Interest Group and Evidence-based Healthcare Implementation interest groups of the Society for Academic Emergency Medicine. In their study, the authors identified the best practices based on a survey of possible scholarly outputs, and only those with high consensus among respondents were considered appropriate scholarship. While the survey respondents were affiliated with most of the national EM groups, representatives of several EM groups, such as the Council of Residency Directors and various EM resident organizations, responded with concern that these recommendations were too stringent and that the approach used to determine the criteria were not consistent with widely accepted definitions of consensus.^7^ They recommended using a less strict consensus threshold, which would result in a more varied list of acceptable scholarship opportunities.

Understanding the limitations of the consensus, it is not surprising that given our inclusion of non-traditional and more modern forms of scholarship (eg, online educational resources, participation in national committees, or FOAMed), we did not see a significant increase in those types of scholarships only noted in the consensus document. It is certainly possible that the lack of increase indicates a lack of impact of our scholarly activity guideline, but we feel it more reflects the bias of the consensus guideline toward more traditional research activities. For example, in the post-SAG group, we had residents pursue podcasts, evidence-based reviews, and participate in national committees, all types of scholarship we feel are valuable that would not have otherwise been counted in the consensus guideline.

There are several other potential explanations for the small change seen in our analysis. Even with the addition of diverse faculty and increasing the focus on resident scholarship, our research infrastructure has remained minimal, which we feel has slowed the advancement of our research mission and potentially limited the potential of the point system. The majority of our residents also become community emergency physicians and are not necessarily focused on research, similar to trainees in many EM residencies. While we did not analyze the points from residents who pursued community practice against those who pursued fellowships or academic medicine, we suspect that those entering community emergency departments likely chose less traditional research and may have been more likely to finish with the minimum points required. If true, this would not necessarily be a negative reflection of the point system; rather, for us it would highlight the flexibility of this approach to all types of residents. We never expected that all our residents would suddenly engage in complex scholarship, but we did hope for an explicit guideline, a clear minimum standard for completion and, overall, more high quality output. We still see our improvements, while modest from a statistical standpoint, as a major culture shift for our program.

### Next Steps

We believe that future, similar investigations would be beneficial to enhance generalizability of our findings. If other programs were to find success in implementing similar scholarly point systems, especially programs with a variety of resources, we may learn in which settings the point system performs best. Applying this to other groups may also be useful. For example, our program is considering creating a point system for faculty to improve their scholarly output and engagement. While still in the planning stage, this approach could help less research-focused faculty participate in the research mission with a variety of options for compliance.

Based on the experience gained by implementing the SAG, we have realized that documentation is imperative, and suggest having clear, ongoing documentation from the time of idea generation through project completion. This provides transparency for project approval and progression, how many points will be awarded, and when the scholarly work is considered complete. We have found documentation of project status to be most successful in a shared online format with access for the resident, the research committee, and program leadership. Additionally, once a point system is implemented, periodic adjustments are necessary to incorporate new areas of scholarship, or to remove or limit items that may not provide incremental educational benefit to the learner.

## LIMITATIONS

This dataset is limited by recall bias, as there could be projects that were not documented and not available to include in the analysis. While the authors that judged point values were blinded to resident names and graduation year, they may have been able to identify residents’ work based on the project type. Additionally, while the raters had moderately good agreement, they had not previously judged point totals, so these numbers may have been incongruent with the points awarded by the research committee in real time based on knowing the specifics of the project. This is particularly evident when considering that at the time of their graduation, all residents in the post-implementation group were felt to have met the 10-point minimum, yet not all were judged to have met the requirement in this analysis, suggesting that blinding led to underestimation of points scored. However, we felt that creating a blinded review would allow for the most unbiased comparison.

It is also possible that retrospective application of the point system may have overestimated the difference, but we feel this is unlikely. Another limitation is that additional faculty were hired at the time of and following the SAG implementation, and these newer faculty could have improved residents’ abilities to complete high-quality scholarship regardless of guideline implementation. Lastly, while we have found the SAG to be effective in our program, it is possible that, as Wood et al suggest, any initiative that provided enhanced emphasis on resident scholarship could have been equally effective.^10^

## CONCLUSION

The implementation of a point-based scholarly activity guideline improves the quantity, breadth and, in our opinion, the quality of scholarly activity among emergency medicine residents. Given the inclusion of a variety of traditional and non-traditional forms of scholarship and its simplicity, this system could be easily adapted for other EM residencies or any other subspecialty training programs.

## Supplementary Information


