# Supplementary material for: Improvement in Resident Scholarly Output with Implementation of a Scholarly Activity Guideline and Point System
Source: West J Emerg Med. 2023 Aug 15;24(5):861–7. doi: 10.5811/westjem.60346 (PMC10527827; doi:10.5811/westjem.60346)
Supplement: Supplementary file 1 [file wjem-24-861-s001.pdf]

Appendix 1: Scholarly Activity Guideline

XX Department of Emergency Medicine
Residency Scholarly Activity Guidelines
Accepted May 20, 2014

Background

ACGME rules require the following for emergency medicine residents under section IV.B of the EM Program Requirements:

> **IV.B Residents' Scholarly Activities**
> IV.B.1. The curriculum must advance residents' knowledge of the basic principles of research, including how research is conducted, evaluated, explained to patients, and applied to patient care.
> IV.B.2. Residents should participate in scholarly activity.
> IV.B.3.The sponsoring institution and program should allocate adequate educational resources to facilitate resident involvement in scholarly activities.[1]

While the ACGME mandates scholarly activity, it does not specify what activities qualify as scholarly. This allows residency programs to tailor the scholarly project to the interests of each resident. In parallel with our department's mission, we want to provide our residents with a valuable scholarly project experience. The following guideline has been created by residents and faculty participating in the Research Committee and has been both edited and approved by the chair and the residency director.

What does scholarly activity mean at XX?

There is no universal definition of scholarly activity. Traditionally scholarly work has been equated with research. While original research is an important component of scholarly activity, there are many other ways to be scholarly. Scholarly activity at XX will be defined based on the recommendations of Boyer's 4 components of scholarship as well as the rubric recently proposed by the Accreditation Council for Graduate Medical Education (ACGME), shown below. The four components of scholarship include: Discovery, Integration, Application, and Teaching. [2,3]

TABLE 2 PROPOSED BASELINE RUBRIC FOR ALL ACCREDITATION COUNCIL FOR GRADUATE MEDICAL EDUCATION (ACGME) RESIDENCY REVIEW COMMITTEES (RRCs)

Component of Scholarship	Examples	Assessment Criteria
Discovery = advancing knowledge	Published paper<br>Work resulting in abstract	All 4 components of scholarship should be present when looking at the sum of the core faculty members' work<br>Each resident should be exposed to each of the 4 components of scholarship and should complete at least one scholarly activity during the residency training period<br>More stringent requirements may be instituted by the specialty-specific RRC as needed
Integration = synthesizing knowledge	Case studies or reports<br>Patient education projects	
Application = applying existing knowledge	Participation in national guideline panels<br>Participation in professional societies	
Teaching = disseminating current medical knowledge	Preparing and delivering lecture(s)<br>Curriculum development<br>Development of web-based modules, etc	

Scholarly activity point system

Given that so many diverse activities can be considered scholarly activity, each activity will be assigned points. This point system will ensure all residents are treated equally by

accounting for the fact that different projects will likely require varying amounts of a resident's time and effort. Again, any potential project that is not listed will be considered on a case-by-case basis. Projects with multiple residents participating will likely have the points divided up depending on the amount of work involved.

Table 1. Scholarly Point System*

Type of Scholarly Activity	*Points*
IRB-approved project completed with manuscript submitted to a peer-reviewed journal	≥10
Submission of a manuscript describing a case series, systematic review, or meta-analysis	≥10
Presentation of a poster or oral presentation at a regional, national, or international conference	5
Publication of a book chapter or section	10
Quality-improvement project completed and results shared with peers	7
Initiation of IRB-approved or QI project but project still ongoing at time of graduation	8-10
Submission of a grant for intramural or extramural funding (with IRB approval)	10
Creation and maintenance of an online teaching tool	5
Publication of a letter to the editor in a peer-reviewed medical journal	3-5
Creation of simulation case for simulation curriculum (not published vs published)	3 - 10
Submission to peer-reviewed journal or national conference of a series of interesting cases (ie, visual diagnosis cases or photo competition)	3.5
Publications for the lay public, such as newspaper articles, on medical topics	3
Participation on a national committee	5
Critically appraised topic write-up and submission to journal	5

*This point system was created and published by the Department of Family Medicine and Community Medicine at Eisenhower Army Medical Center[4]. Types of activities and points eligible were edited and tailored to the needs of the XX Department of Emergency Medicine. If projects are submitted that do not fit into one of these categories, the Scholarly Activity Committee will score them individually.

Mandatory minimum for completion of your required scholarly activity

Earn at least 10 points based on the above point system
Attend 1 research meeting per year
Complete departmental EBM modules
Present scholarly work at departmental research day

Timeline for completion of scholarly project

Below is a recommended guideline for residents to follow when completing their required scholarly project. We strongly recommend to plan in a retrograde fashion: for example, choose your deadline for project completion and work backward, creating realistic deadlines for each step in the process.

<u>Intern year</u>

Spring: Identify area of research/scholarly interest and identify potential faculty mentor with similar area of interest

<u>Second year</u>

Summer/fall: Brainstorm project ideas and narrow the project focus. Set a deadline for each step of your project via retrograde planning. Submit project idea form.

Fall: For QI or IRB projects, complete resident scholarly activity protocol form

Fall/early winter: Present research/scholarly activity protocol at the research meeting for approval

Spring: Work out project kinks and initiate project

<u>Third year:</u>

Summer/fall: Finish project

Fall/winter: Document project outcome/results

Spring: Present your scholarly work

Please note: Each resident should have his/her project approved by the Research Committee before the end of second year.

Who to contact with questions:

XX - Chair of Research Committee
XX - Residency Director

References

1. ACGME Program Requirements for Graduate Medical Education in Emergency Medicine. Accreditation Council for Graduate Medical Education, 2012. at https://http://www.acgme.org/acgmeweb/Portals/0/PFAssets/2013-PR-FAQ-PIF/110_emergency_medicine_07012013.pdf.)
2. Boyer EL. Scholarship Reconsidered: Priorities of the Professoriate. Princeton, NJ: Carnegie Foundation for the Advancement of Teaching, 1990.
3. Grady EC, Roise A, Barr D, et al. Defining scholarly activity in graduate medical education. Journal of graduate medical education 2012;4:558-61.
4. Seehusen DA, Asplund CA, Friedman M. A point system for resident scholarly activity. Family medicine 2009;41:467-9.

XX Resident Scholarly Activity Idea Form

Resident Name ____

Faculty Mentor ____

Area of interest ____

Type(s) of scholarly activity ____

____

____

Research question____

____

____

Will you need:

IRB approval? YES NO

Grant support? YES NO

Statistical support? YES NO

Expected points earned ___________

Please describe your general project timeline below.

____

____

____

____

____

____

____
